# When Zoonosis Meets Genetics: Brucellosis and Gilbert Syndrome in a Young Woman From Rural Nepal

**DOI:** 10.7759/cureus.89608

**Published:** 2025-08-08

**Authors:** Rayana Shrestha, Nava R Sharma, Abhishesh Wagle, Madalasa Pokhrel

**Affiliations:** 1 Department of Internal Medicine, Institute of Medicine, Maharajgunj, Kathmandu, NPL; 2 Department of Internal Medicine, Maimonides Medical Center, Brooklyn, USA; 3 Department of Medicine, Manipal College of Medical Sciences, Pokhara, NPL

**Keywords:** brucellosis, brucellosis complications, cholestatic jaundice, gilbert syndrome, remote nepal, zoonotic infection

## Abstract

Brucellosis is a neglected zoonotic infection in Nepal that is often underdiagnosed, particularly in regions considered to have low prevalence. Its presentation can mimic autoimmune or hepatic disorders, complicating timely diagnosis and management. Coexistence with hereditary conditions such as Gilbert syndrome can further obscure the clinical picture. We present a case of a 28-year-old woman who presented with progressive jaundice, low-grade fever, arthralgia, and weakness. History revealed recurrent jaundice and similar symptoms in family members. The exam showed scleral icterus and hepatosplenomegaly. Labs indicated anemia, leukopenia, and unconjugated hyperbilirubinemia with normal liver enzymes. Brucellosis was confirmed by positive serology and treated with doxycycline and rifampicin, resolving systemic symptoms. Persistent hyperbilirubinemia and family history suggested underlying Gilbert syndrome. This case highlights the diagnostic complexity when infectious and hereditary causes of jaundice coexist. It emphasizes the need for heightened clinical suspicion for brucellosis even in regions of Nepal not traditionally recognized as endemic. A systematic diagnostic approach is essential in patients with unexplained jaundice, particularly when common infectious and autoimmune causes are excluded.

## Introduction

Brucellosis is a globally prevalent zoonotic infection that poses a significant public health challenge, particularly in developing countries where close contact between humans and livestock is common. In Nepal, where animal husbandry forms the foundation of rural livelihoods, brucellosis remains an underrecognized threat despite clear evidence of endemicity. The disease was first documented in Nepal in 1979, when *Brucella* species were isolated from a shepherd in Pokhara [1]. A considerable burden of disease may be underdiagnosed due to non-specific clinical features and limited diagnostic access, especially in rural and remote regions [1]. According to the World Health Organization, more than 500,000 new human cases are estimated to occur globally each year, primarily in the Mediterranean basin, the Middle East, South Asia, and Latin America [1].

Clinically, brucellosis presents with a broad spectrum of manifestations, including fever, malaise, arthralgia, hepatomegaly, and splenomegaly. Hepatic involvement occurs in a substantial number of patients [2]. In some cases, laboratory findings such as elevated liver enzymes or jaundice may mimic viral hepatitis or autoimmune liver disease, complicating diagnosis. The pathophysiology of brucellosis involves intracellular survival of *Brucella* species within macrophages, enabling the organism to evade immune responses and cause chronic granulomatous inflammation. This can affect multiple organ systems, including the liver, spleen, bone marrow, and joints [1,2]. In young women, systemic features such as arthralgia, anemia, and leukopenia may resemble autoimmune conditions such as systemic lupus erythematosus, increasing the risk of misdiagnosis.

Gilbert syndrome is a benign hereditary disorder that causes intermittent unconjugated hyperbilirubinemia due to decreased activity of the enzyme uridine 5'-diphospho (UDP)-glucuronosyltransferase [3]. It affects approximately 5-10% of the general population and is usually asymptomatic. Gilbert syndrome follows an autosomal recessive inheritance pattern and is most commonly caused by a mutation in the promoter region of the UGT1A1 gene, such as the A(TA)7TAA variant, which reduces transcription and enzymatic activity [3]. Although it is not typically associated with significant morbidity, bilirubin levels can rise in response to stress, fasting, or acute illness. When this genetic condition coexists with infections such as brucellosis, it may complicate the diagnostic picture by obscuring the etiology of jaundice.

We present a case of brucellosis in a young woman from western Nepal with persistent jaundice attributed to underlying Gilbert syndrome, highlighting the diagnostic complexity in resource-limited settings.

## Case presentation

A 28-year-old woman from Baglung district presented to the medical team with a seven-day history of progressive jaundice accompanied by decreased appetite and generalized weakness. Her symptoms had evolved over the preceding two days to include subjective fever and diffuse joint and muscle pain. The patient's medical history was remarkable for multiple episodes of self-resolving jaundice, with several family members experiencing similar recurrent episodes.

Physical examination revealed a mildly ill-appearing woman with stable vital signs except for the low-grade fever. The most striking findings included obvious icterus of the sclera and skin, along with palpable hepatosplenomegaly on abdominal examination. No lymphadenopathy was detected, and cardiovascular and respiratory examinations were unremarkable.

The initial clinical presentation created a diagnostic challenge. The combination of young age, female gender, fever, arthralgia, and what would soon be confirmed as leukopenia and anemia raised suspicion for autoimmune conditions as the primary differential diagnosis. However, the presence of hepatosplenomegaly and the patient's rural background broadened the differential to include various infectious etiologies. Laboratory investigations revealed significant abnormalities that deepened the diagnostic complexity. The detailed hematological parameters are presented in Table 1.

**Table 1:** Hematological parameters.

Parameter	Initial result	Follow-up result	Reference range
Hemoglobin (Hb)	8.6 g/dL	10.4 g/dL	13–17 g/dL
Total leukocyte count (TLC)	1100/μL	4600/μL	4000–11000/μL
Neutrophils (%)	54%	75%	40–75%
Lymphocytes (%)	37%	20%	20–45%
Eosinophils (%)	2%	3%	1–6%
Monocytes (%)	6%	2%	2–10%
Basophils (%)	1%	0%	0–1%
Platelet count	250 × 10³/μL	204 × 10³/μL	150–400 × 10³/μL
Packed cell volume (PCV)	26%	28%	35–55%
Erythrocyte sedimentation rate (ESR)	29 mm/hr	–	<20 mm/hr
Mean corpuscular volume (MCV)	80 fL	89 fL	82–92 fL

The hematological studies revealed anemia with hemoglobin of 8.6 g/dL, pronounced leukopenia with a total count of 1,100 cells/μL, and a normal platelet count. The leukopenia was characterized by relative lymphocytosis (37%) and neutropenia (54%). Biochemical analysis demonstrated marked hyperbilirubinemia with total bilirubin of 9.2 mg/dL, predominantly unconjugated (indirect bilirubin = 7.9 mg/dL), while liver enzymes remained within normal limits, a pattern suggesting either hemolysis or conjugation defects rather than hepatocellular injury. The biochemical parameters are presented in Table 2.

**Table 2:** Biochemical parameters. ALT: alanine aminotransferase; AST: aspartate aminotransferase.

Parameter	Initial result	Follow-up result	Reference range
Random blood sugar (RBS)	98	101 mg/dL	60–140 mg/dL
Blood urea nitrogen (BUN)	15 mg/dL	27 mg/dL	15–45 mg/dL
Serum creatinine	0.5 mg/dL	1.4 mg/dL	0.4–1.4 mg/dL
Sodium	140 mEq/L	145 mEq/L	135–155 mEq/L
Potassium	4.2 mEq/L	3.9 mEq/L	3.5–5.5 mEq/L
Total bilirubin	9.2 mg/dL	3.3 mg/dL	0.3–1.4 mg/dL
Direct bilirubin	1.3 mg/dL	0.5 mg/dL	0.0–0.4 mg/dL
Alkaline phosphatase (ALP)	107 U/L	61 U/L	53–128 U/L
Serum glutamic pyruvic transaminase (SGPT/ALT)	20 U/L	14 U/L	Up to 42 U/L
Serum glutamic oxaloacetic transaminase (SGOT/AST)	18 U/L	23 U/L	Up to 37 U/L
Ferritin	328.80 ng/mL	–	13–400 ng/mL

The systematic approach to investigating her jaundice began with screening for common causes. Hepatitis A, B, C, and E serologies were negative, effectively excluding viral hepatitis. Autoimmune markers, including antinuclear antibodies (ANA), returned negative, gradually shifting focus away from autoimmune etiologies despite the compelling clinical presentation. Additional investigations for malaria and typhoid fever also yielded negative results.

Given the epidemiological context of rural Nepal, where livestock farming is prevalent, and the systematic exclusion of common causes of jaundice, the medical team considered zoonotic infections in their differential diagnosis. Despite the relative rarity of reported brucellosis cases in Baglung district, the combination of fever, arthralgia, hepatosplenomegaly, and the patient's rural background prompted *Brucella* antibody testing. The positive result with a titer of 1:40 confirmed the diagnosis and provided the missing piece of the diagnostic puzzle.

Treatment was initiated with the standard regimen for uncomplicated brucellosis with oral doxycycline 100 mg twice daily and rifampicin 600 mg once daily. The patient's response was remarkable, with resolution of fever and joint pain within the first week of treatment. Her energy levels improved significantly, and follow-up imaging confirmed the gradual resolution of hepatosplenomegaly.

Laboratory follow-up revealed improving trends: hemoglobin rose to 10.4 g/dL and total leukocyte count normalized to 4600/μL. Platelet count remained stable. Biochemically, total bilirubin decreased to 3.3 mg/dL but remained elevated, with persistently high indirect bilirubin (2.8 mg/dL) and normal liver enzymes (alanine aminotransferase = 14 U/L; aspartate aminotransferase = 23 U/L). Renal parameters also showed mild elevation in serum creatinine to 1.4 mg/dL, which remained within upper normal limits.

Despite resolution of systemic symptoms and hepatic enlargement, the persistent unconjugated hyperbilirubinemia prompted further consideration of hereditary causes. Combined with her personal and family history of recurrent jaundice episodes, this finding strongly suggested the presence of Gilbert syndrome as an underlying condition that had been unmasked or exacerbated by the acute brucellosis infection.

## Discussion

This case illustrates the diagnostic complexity that can arise when uncommon infections present with overlapping clinical features of more common conditions. The initial presentation raised suspicion for autoimmune conditions, a consideration that would have been reasonable given the patient's demographics and symptom complex. However, the systematic approach to diagnosis and the consideration of the epidemiological context ultimately led to the correct identification of brucellosis.

The regional distribution of brucellosis in Nepal shows significant variation, with higher prevalence reported in areas with intensive livestock farming [1]. Western Nepal, including Baglung district, has historically reported fewer cases compared to other regions, making this diagnosis less obvious. However, recent studies suggest that brucellosis may be more widespread than previously recognized, with underdiagnosis and underreporting contributing to the apparent regional variations [1,4].

The hepatic manifestations of brucellosis are well documented, with studies showing that hepatomegaly occurs in 40-60% of cases, splenomegaly in 30-50%, and jaundice in 10-20% of patients [5-7]. The mechanism of hepatic involvement in brucellosis is multifactorial, including direct bacterial invasion, immune-mediated inflammation, and granulomatous hepatitis [6-9]. In this case, the predominant unconjugated hyperbilirubinemia with normal liver enzymes suggested a different mechanism, likely related to hemolysis or the underlying Gilbert syndrome [3,9].

The coexistence of Gilbert syndrome with acute brucellosis created a unique clinical scenario. Gilbert syndrome, caused by reduced UDP glucuronosyltransferase activity, can be exacerbated by various stressors, including infections, fasting, and physical stress [10]. The acute brucellosis infection likely precipitated a more severe episode of jaundice due to the underlying enzymatic deficiency, complicating the clinical picture and potentially masking the infectious etiology.

The diagnostic approach in this case highlights several important considerations for healthcare providers in endemic regions. First, the importance of maintaining high clinical suspicion for zoonotic diseases, even in areas where they are considered uncommon. Second, the need for systematic evaluation when common causes of jaundice are excluded. Third, the recognition that multiple conditions can coexist and complicate clinical presentations.

The treatment response validated the diagnosis and demonstrated the effectiveness of standard antibiotic therapy for brucellosis. The persistence of mild unconjugated hyperbilirubinemia after successful treatment of brucellosis, combined with the family history, strongly supported the diagnosis of Gilbert syndrome. This finding emphasizes the importance of considering hereditary causes of hyperbilirubinemia, particularly when jaundice persists despite treatment of the acute condition.

This case highlights how overlapping clinical features of infectious and genetic conditions can obscure diagnosis, particularly in resource-limited settings. The concurrent presentation of brucellosis and Gilbert syndrome led to diagnostic uncertainty, underscoring the need for broad differentials and awareness of regional disease epidemiology. In regions where brucellosis is often overlooked, especially among female patients with nonspecific systemic symptoms, this case reinforces the importance of considering zoonotic diseases. Furthermore, it serves as a reminder that genetic conditions like Gilbert syndrome, though benign, can unmask or mimic other disease processes. Clinicians should maintain a high index of suspicion for dual pathology in patients who do not fit a typical disease pattern or fail to fully improve despite targeted treatment.

## Conclusions

The convergence of brucellosis and Gilbert syndrome in this patient from western Nepal created a diagnostic challenge that required careful clinical reasoning and systematic investigation. The case demonstrates that uncommon infections can present with common symptoms, and that epidemiological context remains crucial in guiding diagnostic decisions. The successful identification and treatment of brucellosis, combined with the recognition of underlying Gilbert syndrome, highlights the importance of considering multiple conditions when clinical presentations do not fit typical patterns.

This case contributes to the growing body of literature documenting brucellosis in western Nepal and emphasizes the need for continued vigilance for zoonotic diseases throughout the country. Healthcare providers should maintain high clinical suspicion for brucellosis when evaluating patients with unexplained fever, arthralgia, and hepatosplenomegaly, even in regions where the disease is considered uncommon. The coexistence of hereditary conditions like Gilbert syndrome with acute infections serves as a reminder that multiple pathological processes can occur simultaneously, requiring comprehensive evaluation and individualized treatment approaches.
